# Antibody Mediated Transduction of Therapeutic Proteins into Living Cells

**DOI:** 10.1100/tsw.2005.98

**Published:** 2005-09-16

**Authors:** James E. Hansen, Richard H. Weisbart, Robert N. Nishimura

**Affiliations:** ^1^ 1Department of Medicine – San Fernando Valley Program, University of California, Los Angeles, CA 90095, USA; ^2^ Department of Research, Veterans Affairs Greater Los Angeles Healthcare System, Sepulveda, CA 91343, USA; ^3^ Department of Neurology, David Geffen School of Medicine, University of California, Los Angeles, CA 90095, USA

**Keywords:** antibody, antibodies, autoantibody, autoantibodies, single-chain Fv, protein therapy, gene therapy, p53, dystrophin, cancer, muscular dystrophy, protein transduction, ptd, Tat, penetratin, Herpes Simplex VP22

## Abstract

Protein therapy refers to the direct delivery of therapeutic proteins to cells and tissues with the goal of ameliorating or modifying a disease process. Current techniques for delivering proteins across cell membranes include taking advantage of receptor-mediated endocytosis or using protein transduction domains that penetrate directly into cells. The most commonly used protein transduction domains are small cell-penetrating peptides derived from such proteins as the HIV-1 Tat protein. A novel protein transduction domain developed as the single chain fragment (Fv) of a murine anti-DNA autoantibody, mAb 3E10, has recently been developed and used to deliver biologically active proteins to living cells *in vitro*. This review will provide a brief overview of the development of the Fv fragment and provide a summary of recent studies using Fv to deliver therapeutic peptides and proteins (such as a C-terminal p53 peptide, C-terminal p53 antibody fragment, full-length p53, and micro-dystrophin) to cells.

## INTRODUCTION

Development of a means to restore missing proteins to diseased cells consistently and efficiently remains a prominent goal in molecular medicine. While gene therapy has the potential to accomplish this task, considerable obstacles must be overcome before it becomes clinically applicable. Alternatives to gene therapy need to be considered in the interim. One such alternative, protein therapy, comprises the direct delivery of missing proteins to cells. Protein therapy circumvents the need for transfer of genetic material into cells, thereby avoiding many of the problems and dangers associated with gene therapy.

As protein transduction techniques improve, greater interest in protein therapy has driven increasing efforts toward discovering and refining protein delivery vehicles. A significant amount of research has focused on cell-penetrating peptides (CPPs), which are short, basic peptides that penetrate into living cells[1]. The most frequently studied CPPs include HIV-1 Tat peptide, HSV VP-22 peptide, penetratin, and simple polyarginine peptides. Fusion of CPPs to other proteins confers transducibility to the fusion protein and has been used to deliver a variety of proteins to living cells both *in vitro* and *in vivo*[2,3,4,5,6]. While these peptides are of obvious interest and hold great potential in protein therapy, their use in the treatment of human disease may be limited by documented proinflammatory and toxic characteristics[7,8,9,10].

## ANTIBODIES AS INTRACELLULAR DELIVERY VEHICLES

Receptor-mediated endocytosis of antibody-conjugated molecules has been extensively studied and reviewed[11]. The use of antibodies and antibody fragments as protein transduction domains, however, is an emerging field. Living cells can be penetrated by certain autoantibodies, including some that bind DNA[12,13]. The penetration of living cells by these anti-DNA autoantibodies led researchers to investigate the potential of autoantibody protein transduction domains as intracellular protein delivery vehicles. The noninflammatory and nonimmunogenic nature of antibodies coupled with their potential to induce tolerance to themselves and to a protein cargo make antibodies highly attractive candidates for development into intracellular delivery vehicles[14,15]. While many autoantibodies have associated cytotoxicities, some appear to be benign. An anti-DNA autoantibody produced by MRL/mpj/lpr mice with lupus nephritis, mAb 3E10, has recently been developed into a novel intracellular delivery vehicle and has been used to deliver a multitude of potentially therapeutic proteins into living cells[16]. This review will provide a brief survey of recent progress that has been made in using mAb 3E10 to deliver therapeutic proteins to living cells.

## mAb 3E10

In 2000, Weisbart et al. demonstrated a novel role for mAb 3E10 in delivering functional proteins to living cells. mAb 3E10 was covalently linked to bovine catalase enzyme, and the conjugate protein was analyzed both for ability to penetrate into living cells *in vitro* and to protect primary rat cortical neurons against oxidative damage. Neurons incubated with the mAb 3E10-catalase conjugate showed penetration of the conjugate into both the nuclei and cytoplasm of the neurons, while neurons incubated with free catalase showed no uptake of catalase into the cytoplasm or nuclei. Additionally, neurons transduced by the mAb 3E10-catalase conjugate were resistant to $H_2O_2$ toxicity compared to neurons incubated with unconjugated catalase[17]. The results demonstrated that mAb 3E10 was not only capable of penetrating into living cells itself, but also of carrying another protein into cells. Furthermore, the neuroprotection afforded by the mAb 3E10-catalase conjugate protein established the ability of mAb 3E10 to deliver functional, biologically active proteins to living cells.

Additional efforts focused on optimizing the transduction efficiency of the mAb 3E10 antibody and identifying the minimal portions of the antibody required for cellular penetration. First, a single change of an aspartic acid to an asparagine residue at position 31 in CDR 1 of the $V_H$ region of the antibody greatly increased its transduction efficiency as indicated by greater percentages of cells transduced at smaller concentrations of the antibody[16]. Second, smaller Fab and single-chain fragments of the mutant mAb 3E10 antibody were found to penetrate into living cells equally as well as the full-length antibody. Impressively, Fab fragments delivered alkaline phosphatase-conjugated goat antibodies, estimated at 305 kDa in size, into the nuclei of living cells[18]. While the full-length mAb 3E10 and Fab fragments of mAb 3E10 have been established as viable intracellular delivery vehicles for protein transduction, the single-chain fragment has been the primary focus of recent studies.

## THE FV FRAGMENT OF MAB 3E10

The single-chain fragment of mAb 3E10, Fv, consists of the variable region of both the light and heavy chains of mAb 3E10 cross-linked by a short $(GGGGS)_3$ linking sequence (Fig. 1). Fv penetrates living cells and localizes to the nucleus as well as the full-length antibody and the Fab fragments. Fv-fusion proteins also penetrate into cells and nuclei. For example, an Fv-GFP fusion protein was the first Fv-fusion protein studied, and it entered cells and localized to nuclei[18]. The smaller size of the Fv fragment when compared to the mAb 3E10 antibody or its Fab fragments makes Fv most attractive in attempting to deliver therapeutic proteins to cells. Toxicity of the Fv fragment has been excluded by generation of CHO cell lines stably expressing Fv cDNA. These CHO cells were subjected to Fv in two ways: they synthesized and secreted the Fv fragment and were then transduced by the secreted Fv. The cells proliferated at a normal rate and excluded trypan blue comparably to other nontransfected CHO cells[18]. After the identification of the Fv fragment as a small component of the antibody capable of transduction and with its demonstrated absence of cytotoxicity, several studies successfully used the Fv fragment to deliver therapeutic peptides and proteins to cells.

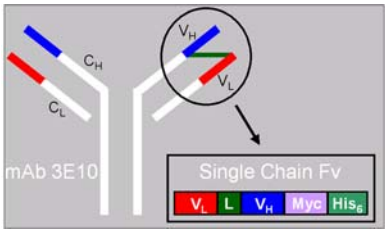

**FIGURE 1.** The single-chain Fv fragment of mAb 3E10. A transducible single-chain Fv fragment of mAb 3E10 was generated by cross-linking the variable regions of the antibody's light ($V_L$) and heavy ($V_H$) chains. A myc tag was added to aid in detection of the Fv fragment and a histidine tag was added for purification of the Fv fragment and Fv-fusion proteins. (L = $[GGGGS]_3$ linking sequence, $C_H$ = constant region of mAb 3E10 heavy chain, $C_L$ = constant region of mAb 3E10 light chain.)

## FV AS A MEANS TO RESTORE P53 FUNCTION TO CANCER CELLS

Of the cancers afflicting humans worldwide, approximately 50% are related to mutations or defects in the p53 tumor suppressor protein, and a significant amount of effort has been focused into restoring functional p53 to cancer cells[19,20,21]. At least part of p53 activity depends on its ability to activate transcription of specific target genes, and given Fv's predilection for localizing to cell nuclei, Fv is uniquely positioned to deliver factors into the nucleus of cancer cells with the goal of modulating p53 activity. Several reported studies have established that C-terminal peptides of p53 and antibodies that bind the C-terminus of p53 activate transcription-defective mutant p53[22,23]. However, these peptides and antibodies lack an efficient means of delivery into cells. Fv was examined for its ability to deliver such C-terminal peptides or antibodies to cancer cells in the hopes of activating mutant p53 and killing cancer cells.

A fusion protein consisting of the Fv fragment and a 30-mer C-terminal p53 peptide known to activate mutant p53 was produced as a secreted protein in Cos-7 cells. SW480 human colon adenocarcinoma cells were selected for testing the Fv-peptide fusion protein, as these cells are cancerous due to a mutation in p53 that renders it transcriptionally inactive. The Fv-peptide fusion protein penetrated into the SW480 cells and localized to nuclei as expected, but induced only modest killing of the cancer cells (approximately 10% of cells). In an assay to detect transcriptional activity of p53, SW480 cells were transfected with a CAT reporter plasmid. As expected, control, nontransduced SW480 cells showed no baseline p53 trancriptional activation. Interestingly, SW480 cells transduced by the Fv-peptide fusion protein also failed to report any detectable level of p53 transcriptional activity[24]. The failure of the Fv-peptide fusion protein to induce any detectable level of activation of p53 likely accounts for the low percentage of cells killed, and may indicate that the comparatively large Fv fragment interfered with the ability of the peptide to interact with p53.

Next, a bispecific antibody composed of the Fv fragment and a single-chain fragment of an anti-p53 antibody, mAb PAb421, was constructed and expressed as a secreted protein in *Pichia pastoris*. Similar to the C-terminal peptides of p53, mAb PAb421 binds the C-terminus of p53 and restores transcriptional activity to mutant p53[25,26]. Two cell lines, SW480 and Cos-7 African green monkey kidney cells, were used to test the bispecific antibody. SW480 cells, with their mutant p53, would be expected to be sensitive to activation of p53 by mAb PAb421. Cos-7 cells, which express the SV40 large T antigen that prevents the mAb PAb421 antibody from binding the C-terminus of p53, should be resistant to activation of p53 by the bispecific antibody. The bispecific antibody penetrated into both cell types and, as predicted, induced cell death in the SW480 cells but spared the Cos-7 cells. While the bispecific antibody appeared to specifically activate mutant p53 in the SW480 cells and induce cell killing, the relative amount of cell death was only 36%. The modest level of killing by the bispecific antibody was attributed to low levels of mutant p53 in the SW480 cells. Transfection of a plasmid to induce increased expression of p53 into cells prior to treatment with the bispecific antibody increased the percentage of cells killed to approximately 55%[27].

## FV DELIVERS FUNCTIONAL, FULL-LENGTH P53 TO CANCER CELLS

Given the results achieved with both the Fv-peptide fusion protein and the bispecific antibody, attention shifted from using Fv as a means to activate mutant p53 to using Fv to deliver full-length, functional wild type p53 protein to cancer cells. An Fv-p53 fusion protein was generated and expressed as an intracellular protein in *P. pastoris*. Several cell lines were used to test the efficacy of Fv-p53, including wild type primary cells (primary astrocytes) and cells cancerous due to absence of p53 (Saos-2 human osteosarcoma and Skov-3 human ovarian carcinoma cells), mutation in p53 (HT-29 human colon adenocarcinoma cells), nuclear exclusion of p53 (PC-12 human pheochromocytoma cells), overexpression of the negative regulatory protein MDM2 (MC-7 human breast cancer cells), and expression of T antigen (Cos-7 cells). Fv-p53 successfully penetrated into all of the cells examined and localized in both the nuclei and throughout the cytoplasm. Excitingly, Fv-p53 had a profound and specific effect on the cells, killing approximately 90% of the Saos-2, Skov-3, PC-12, and MC-7 cells and 45% of the HT-29 cells, while sparing the primary astrocytes and Cos-7 cells[28]. Whether Fv-p53 induces apoptosis in cancer cells through transcription-dependent or transcription-independent pathways is presently under investigation. Regardless of the mechanism, Fv-p53 is effective in penetrating and killing a vast array of cancer cell types, while at the same time sparing normal control cells, indicating great clinical potential in the treatment of cancer. The true clinical potential of Fv-p53 is currently being evaluated in *in vivo* experiments.

## FV DELIVERS MICRODYSTROPHIN TO COS-7 CELLS, MYOBLASTS, AND MYOTUBES

Duchenne muscular dystrophy is an X-linked recessive neuromuscular disease caused by deficiency in the protein dystrophin, a key member of the dystrophin-associated protein complex in the sarcolemma. Dystrophin binds to and links the transmembrane protein β-dystroglycan with the actin cytoskeleton in skeletal muscle. In the absence of dystrophin, muscle cells undergo repeated cycles of regeneration and atrophy. Multiple studies have demonstrated that restoration of dystrophin to skeletal muscle via transgene expression or viral-mediated gene therapy ameliorates the disease phenotype[29]. However, safe and efficient means to deliver the dystrophin gene to dystrophin-deficient muscle in a clinical setting have not yet materialized.

The Fv fragment was tested as a means to deliver dystrophin protein directly to muscle cells, circumventing the need for gene therapy. Because full-length dystrophin is a large protein, 427 kDa in size, a microdystrophin protein was selected for fusion to the Fv fragment. Microdystrophin is a truncated version of the dystrophin protein that has been shown sufficient to prevent the dystrophic phenotype[30]. An Fv-microdystrophin (Fv-MD) fusion protein produced as an intracellular protein in *P. pastoris* was tested on L6 myoblasts and myotubes and Cos-7 cells. Fv-MD penetrated into the L6 cells and localized throughout the cytoplasm and nuclei, indicating that Fv is capable of delivering proteins to muscle cells[31].

More interestingly, Fv-MD was observed to penetrate into and localize to the periphery of Cos-7 cells, apparently interacting with the cell membrane. Full-length, wild type dystrophin expressed as a transgene in Cos-7 cells also localized to the membrane of Cos-7 cells[32]. It is proposed that, similar to its behavior in muscle cells, dystrophin interacts with β-dystroglycan in the membrane of the Cos-7 cell, causing it to localize to the membrane. Staining for the presence of β-dystroglycan in the Cos-7 cells and the L6 cells used to test the Fv-MD revealed that only the Cos-7 cells possessed β-dystroglycan in their membranes. The immature L6 cells were found to have β-dystroglycan localized in a primarily perinuclear pattern. This difference in distribution of β-dystroglycan not only explains the different localization of Fv-MD in Cos-7 cells vs. L6 cells, but also suggests that Fv-MD is capable of interacting with β-dystroglycan at the Cos-7 cell membrane and therefore possesses at least some biological activity[31]. Further experiments *in vivo* are needed to determine Fv-MD's potential as a clinical therapy for Duchenne muscular dystrophy.

## CONCLUSION

mAb 3E10 and its Fv fragment are unique among all other protein transduction domains. The documented nuclear localization of mAb 3E10 and Fv makes them ideal agents for intranuclear delivery of proteins such as transcription factors. In a scientific era in which manipulation of gene expression is becoming increasingly relevant both *in vitro* and *in vivo*, a benign intranuclear delivery vehicle is highly desirable. An increasing number of investigators have attempted to use the HIV-1 Tat CPP to deliver specific transcription factors to stem cells in the hopes of driving their differentiation in a specific direction, and it is likely that Fv, with its intrinsic tendency to localize to the nucleus, could be highly successful in accomplishing such a task[33,34].

In addition to delivering peptides and proteins to nuclei, it is also apparent from recent studies that Fv can deliver proteins to the cytoplasm. The precise mechanism by which Fv enters into cells and then localizes into the nuclei or cytoplasm is presently unknown. However, there is likely an interaction between Fv and the cargo protein it carries into the cell that ultimately determines localization of the fusion protein. The ability to deliver proteins both to the nucleus and the cytoplasm of a cell makes the Fv fragment a diverse delivery vehicle, ideal for delivering proteins that may have both nuclear and cytoplasmic roles. For example, efforts are underway to develop an Fv-Hsp70 fusion protein as a potential therapy for ischemic stroke and myocardial infarction. The Fv fragment could also be used to deliver impermeable intrabodies into living cells[35,36].

The use of an antibody-derived protein transduction domain in protein therapy is a plausible alternative to gene therapy and the CPPs. Replacement of a protein to cells and tissues in an organism which has never before encountered the protein is likely to result in the generation of an immune response to a variety of antigens on the missing protein. It is possible that mAb 3E10 or its Fv fragment will induce immune tolerance to themselves and to any protein cargo they carry into cells. mAb 3E10 and its Fv fragment have been clearly established as an effective means of delivering functional proteins to living cells *in vitro*. If they should prove equally as effective *in vivo*, they will have a profound impact on the clinical viability of protein therapy for diseases caused by deficiency in a single protein and will have significant applications in the regulation of nuclear processes.
